# Factors associated with the identification of child mental health problems in primary care—a systematic review

**DOI:** 10.1080/13814788.2019.1623199

**Published:** 2019-06-27

**Authors:** Nynke R. Koning, Frederike L. Büchner, Marjolein E.A. Verbiest, Robert R.J.M. Vermeiren, Mattijs E. Numans, Mathilde R. Crone

**Affiliations:** a Department of Public Health and Primary Care, Leiden University Medical Centre, Leiden, The Netherlands;; b Centre for Longitudinal Research-He Ara ki Mua, The University of Auckland, Auckland, New Zealand;; c National Institute for Health Innovation, The University of Auckland, Auckland, New Zealand;; d Department of Child and Adolescent Psychiatry, Leiden University Medical Centre, Curium-LUMC, Oegstgeest, The Netherlands;; e Department of Child and Adolescent Psychiatry Amsterdam, UMC location VU, Amsterdam, The Netherlands

**Keywords:** Children, mental health, primary care

## Abstract

**Background:** Although common and often with long-lasting effects, child mental health problems (MHPs) are still under-recognized and under-treated. A better understanding of the factors associated with the identification of MHPs in primary care may improve the recognition of MHPs.

**Objectives:** To review studies on factors associated with the identification of child MHPs in primary care.

**Methods:** Six leading databases were systematically searched until 1 October 2018. Two independent researchers selected articles and extracted data on study characteristics and factors associated with MHP identification. Inclusion criteria were the investigation of factors associated with MHP identification by primary care professionals (PCPs) in children aged 0–18 years.

**Results:** Of the 6215 articles identified, 26 were included. Prevalence rates of PCP-identified MHPs varied between 7 and 30%. PCPs identified 26–60% of children with an increased risk of MHPs as indicated by MHP assessment tools, but associated factors were investigated in relatively few studies. MHPs were more often identified in children with a family composition other than married parents, with worse mental health symptoms, prior MHPs, among boys in elementary school, when contact with PCPs was related to parental psychosocial concerns or routine health check-ups, when PCPs were recently trained in MHPs or when PCPs felt less burdened treating MHPs.

**Conclusion:** MHP identification varied substantially between studies and PCPs and was related to several child, family and practice factors. Future studies should systematically investigate factors associated with MHP identification by PCPs and specifically in children with an increased risk of MHPs according to mental health assessment tools.

KEY MESSAGES

- Identification of child mental health problems (MHPs) varied substantially between studies and professionals.
- MHP identification was related to several child, family and practice factors.
- Future studies should systematically investigate factors associated with PCP identified MHPs, specifically in children with an increased risk of MHPs according to mental health assessment tools.

## Introduction

Mental health problems (MHPs), defined as any emotional, behavioural or developmental problems, are common in children and adolescents [1,2]. The severity of MHPs varies widely, from children with mild problems without impairment, to children with severe impairment [3]. MHPs often have a negative influence on a child’s everyday functioning and well-being and may lead to various adverse outcomes later in life such as a poorer performance at school and/or in the job market and a higher risk of impediment due to a Diagnostic and Statistical Manual of Mental Disorders (DSM) diagnosis later in life [4–10]. Early identification of MHPs in children is thus important to provide adequate treatment strategies and prevent adverse outcomes.

Primary care has a central role in the identification and treatment of children with MHPs [10]. Most countries distinguish primary care professionals (PCPs) who provide preventive care (i.e. preventive youth healthcare focusing on the healthy development of a child) from those PCPs providing curative care (i.e. general practice or paediatric consultation focused on resolving health problems). The majority of children and adolescents in Western societies visit any PCP at least once a year [11–13]. Seeing children regularly throughout childhood, PCPs are in a unique position to manage child MHPs [14]. Governments in developed countries now have a greater awareness of PCPs as the ‘gatekeepers’ of child mental health services [14–17].

Although children regularly visit a PCP, several children will not be recognized as having MHPs [18–21]. For example, in two cohort studies conducted among children visiting a PCP for a routine health assessment in the US and the Netherlands, PCPs did not recognize MHPs in 50% and 43% respectively of the children with high scores on mental health screening tools [22,23]. A potential explanation might be that relevant information is not (explicitly) shared by parents. MHPs in children consequently remain undertreated and a large proportion of children with MHPs do not receive adequate care [24,25].

Over a decade ago, two reviews identified several sometimes contrasting factors associated with identified child MHPs. Both reviews prioritized further research in primary care settings that explored child, parental and service factors influencing primary care identification [25,26]. Since then, new studies regarding the identification of child MHPs in primary care have been conducted. The present study aimed to review systematically the current literature regarding factors associated with PCP identification of child MHPs. In addition, we investigated factors associated with PCP identification of children with an increased risk of MHPs as assessed by MHP screening tools.

## Methods

### Search strategy

We conducted a systematic search for original articles published before 1 October 2018. A search strategy including MeSH terms and broad concepts such as ‘psychosocial problems’ and narrow diagnoses such as ‘anxiety disorder,’ was developed for PubMed and adapted for equivalent searches in Embase, CINAHL, Web of Science, Cochrane and PsycINFO (Supplementary Table 1). In addition, we performed a grey literature search in seven databases (WHO database, OpenGrey, GreyLit, GLIN (Grey Literature in the Netherlands), Academic Search Premier, Clinical Trials and Current Controlled Trials) to avoid missing relevant titles published outside the conventional databases.

### Inclusion and exclusion criteria

The title and abstract and after that the full text of the articles were independently screened by two authors (NK and FB) using predefined inclusion and exclusion criteria. We included studies that: (1) focused on children aged 0–18 years who visited a PCP (directly or indirectly through parents or caretakers), (2) examined PCP-identified MHPs, and (3) explored factors associated with identified MHPs. We defined MHPs as any emotional, behavioural or developmental problem causing mild to severe impairment. Exclusion criteria were: (1) articles that contained non-original data, (2) reviews, dissertations, book chapters, case reports, editorials, oral presentations and poster presentations, and (3) articles published in a language other than English or Dutch.

### Quality appraisal

Quality assessment of the included studies was performed by a critical appraisal based on standardized criteria using the Crowe Critical Appraisal Tool (CCAT). The CCAT has been tested for validity and reliability [27–30]. Two researchers (NK and MV) appraised the articles independently. Discrepancies in scores were mostly attributable to different interpretations of a sub-item and were discussed in a group meeting with NK, MV and MC until consensus was reached. We did not have a pre-specified CCAT score under which we would exclude a study.

### Data extraction

We extracted general descriptive characteristics from the included studies, as well as factors associated with MHP identification and their effect measures e.g., relative risks or odds ratios. In cases where no effect measure was present, a description of the association between the factor and the outcome was obtained from the text; if this was not reported the study was excluded from further analyses. Unless otherwise specified, only factor associations adjusted for other background variables are presented.

## Results

**Figure 1.**
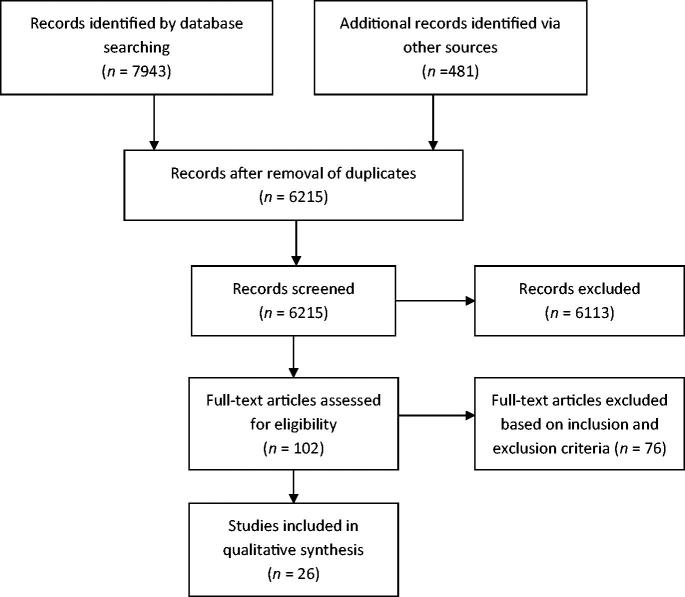
Flow diagram of article inclusion process.

Our initial search resulted in 6215 original titles (Figure 1). Screening of titles, abstracts and full texts resulted in the inclusion of a final set of 26 studies. Reasons for excluding studies were related to a lack of focus on factors associated with PCP identification of MHPs or a study outcome other than PCP-identified MHPs. Quality appraisal scores for the 26 studies ranged from 24 to 33 points (maximum 40), with an average of 27.8 points (Supplementary Table 2). Since we did not assign extremely low or high-quality scores, no studies were excluded from further analysis based on the CCAT.

### General description

The 26 included studies were published between 1992 and 2018 (Supplementary Table 3a). Twelve studies were performed in the US [22,31–41], 11 in the Netherlands [19,20,23,42–49] and three in the UK [21,50,51]. The study setting was general practice in seven studies [19,21,22,36,39,50,51], preventive youth healthcare in 15 [20,23,31,34,37,40–49] and combined preventive youth healthcare and general practice in four studies [32,33,35,38]. All included studies involved cross-sectional analyses of children visiting a PCP. No study included all children in the age range 0–18 years, and most often studies focused on children aged 5–12 years. The studies used different inclusion and exclusion criteria, e.g. regarding age groups, exclusion of children with prior MHPs and acute care visits. Owing to differences between included studies, we present the direction of the associations between investigated factors and the identification of MHPs by PCPs.

MHPs in general (i.e. the broad concept of MHPs) were investigated in 24 studies, mostly by asking the PCP whether MHPs were currently present without defining MHPs specifically [20–23,31–35,37–40,42–50]. One study investigated only depression and anxiety [36], another only depression [51]. Twenty-four studies included information on factors associated with MHPs identified by child, parent and professional questionnaires [19–23,31–40,42–50], sometimes (additionally) by chart review [36,41,51], by interviews with the child/parent [19,23,36,44,45], or by videotape analysis [39]. Thirteen studies compared PCP identification with scores on mental health assessment tools; the results of these studies will be discussed separately [21–23,32,36,38,42,44–46,48–50].

PCPs identified an MHP in 7–30% of children (Supplementary Table 3b). Overall, we found that PCP identification rates were higher in studies that included only preventive care compared to studies that also included curative care.

### Factors associated with PCP identification of MHPs: child characteristics

In children of junior school age (4–12 years), boys were more often identified with MHPs. However, this was not the case in younger or older children (Table 1) [19,23,33,34,36,40,42,44,46,47,49,50]. More MHPs were identified in children with parent-reported problems related to school, and MHPs were also more frequently identified in school-aged children experiencing life events (e.g., divorce) in the past year [23,42,49,51].

**Table 1.** Associations between the investigated factors and PCP identification of mental health problems.

	Factor associated with mental health problem identification^a^	Number of studies	Positive association with identified mental health problems number of studies	Negative association with identified mental health problems number of studies	No association with identified mental health problems number of studies
Child	Higher age	9	5	2, of which 1 study for only age 12–16	2
	Male gender	12	7, of which 1 study only for age 4–11^a^		6, of which 1 only for age 12–17
	Ethnicity	9	Economic immigrant: 1	Black: 1 Hispanic: 1	8, of which 1 specifically for ethnicity former colonies/other (non-) industrialized countries
	Smoker	1	1		
	Alcohol/drugs misuse	1	Alcohol misuse in boys: 1		Drugs misuse: 1
	Life events in past year	4	2		2
	Parent report of school problems	2	2		
	Child-perceived difficulties	2			2
	More visits in past year	4	2		2
Medical history	Neonatal/developmental problems	1			1
	Comorbid conditions	7		1	6
	Somatic complaints	1	1, for e.g. headache, back pain, tiredness		
	Past MHP	1	1		
	Past treatment for MHP	5	General treatment: 3 Psychological treatment: 2 Medical treatment:2 Other treatment: 1		Other treatment: 1
	Child health limitation: parent impression	1	1, only for age 12–17		1, only for age 4–11
Mental health problems based on tool	Child’s MHP: clinical total score	11	10		1
	Child’s MHP: clinical internalizing/emotional symptom score	6	4, of which 1 specifically anxiety/depression symptoms		2
	Child’s MHP: clinical externalizing/behavioural symptom score	5	3		2
	SDQ burden to family	1	1		
	Parent-perceived difficulties (on SDQ)	2	1		1
	Teacher reported MHP on TRF	1	1, only for age 4–11		
Parent/family	Older maternal age	1	1		
	Family structure other than married parents	7	5		2
	Absence of siblings	3			3
	Higher parental education	7		4, of which 1 study only for high level	4, of which 1 study only average level
	Parent unemployed/working <16 h/week	2			2
	Lower socioeconomic status	2		1	1
	Higher area deprivation^b^	2	2		
	Highly urbanized area of home address	2	1		1
	Parental distress	2			2
	Better family functioning	1		1	
	Day care	1		1	
	Parenting practice	1		Overreactive style: 1	Lax style: 1
	Parent sense of competence being parent	2	1		1
	Parent positive affect or negative affect	1			1
	Parent poor MH status/MHP history	2	1		1
Perinatal characteristics	Duration of pregnancy, type of delivery, postdelivery hospitalization of child, birth weight, parity	1			All separately investigated: 1 All investigated together but hospitalization and parity: 1
Professional	Higher age	3			3
	Male gender	2			2
	More work experience	3		> 21 years: 1	3, of which 1 only for <21 years
	Professional training MHP				
	Child well-known	2	2		
	Lower psychosocial orientation	1		1	
	More perceived efficiency treating MHP	1			1
	Lower physician burden	2	1		1
	Physician training in MHP	3	Training 3 months ago: 1		3, of which1 for training 6 months ago
	Job satisfaction	1			1
	Job control	1			1
	Use of screening tool	3	On indication: 1	Always/on indication use of CBCL: 1	Always/on indication use of LSPPK/TRF: 1 Always use of screening instrument: 1
Practice	Practice type (solo/group neighbourhood health centre, prepaid group, multi-specialty)	2			2
	Low accessibility MH specialist	3	1		2
	Composition of practice	1			1
Visit	Type of visit	5	Well-child: 2 Psychosocial: 2		Visit not for MHP: 1
	Season of visit	1			1
	Parent reported discussion MHP	2	1		1
	Physician reported MHP exploration/parental disclosure	3	3		
	Parent initiated disclosure negative psychosocial information (researcher determined)	1	1		
	Parent checklist prompting parental disclosure	1			1
	Longer duration of visit	1	1		

PCP, primary care professionals; LSPPK, national checklist indicating psychosocial problems in 5-year-olds; MH, mental health; MHP, mental health problem; SDQ, strengths and difficulties questionnaire; TRF, teacher report form.

^a^Not included in this table are the associations with identified mental health problems in children with increased scores on mental health problem assessment tools.

^b^Composite, based on postcodes, degree of urbanization, proportion, proportion of ethnic minorities, mean income per earner.

^c^This study presented associations separately for the two age groups 4–11 and 12–17 years [19]; different findings for the different age groups are therefore specified.

Somatic complaints (e.g. headache) and a past (treatment for a) MHP were also related to increased MHP identification, whereas more visits to a PCP in the past year was only related to MHP identification in the case of adolescents [23,31,35,36,42,44,47,49,51]. Neonatal/developmental problems, comorbid conditions, a child’s age or ethnicity were not (consistently) related to MHP identification [19,20,23,31,33–37,40,42–45,47,49–51].

### Characteristics of parent/family

Children with a family structure other than married parents were more often recognized with MHPs in five studies, whereas two studies found no association [23,31,33,34,37,42,47]. MHPs were also more often identified in children living in a deprived area [43,51]. Associations between parental education, socio-economic status, employment status, a family history of MHPs and identified MHPs were inconclusive [19,23,32,33,40,42,44,46,47,49,50]. Other characteristics of the parent/family did not impact MHP identification.

### Professional, practice and visit characteristics

PCP characteristics (e.g., age, gender and work experience) and practice characteristics (e.g. practice type and accessibility of mental healthcare) did not influence PCP identification of MHPs [31,33–35,41,46]. PCPs with less focus on psychosocial well-being identified fewer children with MHPs [33], while PCPs experiencing a lower burden in treating MHPs identified more children [35]. The training of PCPs in MHP identification resulted in increased identification when such training had recently taken place [33,35,48].

Children visiting a PCP for a well-child visit [34,40] or for psychosocial concerns [33,35], and children well-known to a PCP (i.e. the PCP was the child’s usual medical provider), were more often identified with a MHP [33,40]. However, MHPs were more often identified only when PCPs or observers reported discussion of MHPs during consultations. When parents reported discussion or when parents used a checklist to prompt parental disclosure of child MHPs, MHP identification did not increase [21,22,35,39,40,50].

Three studies examined between-professional variance in the identification of child MHPs [37,46,47]. The between-professional variance could not be explained by parent-reported problems [37] or any child-related characteristic [37,46], and could only be partly explained by PCP or practice characteristics [37,46,47].

### Identification of children with an increased risk of MHPs

Thirteen studies compared PCP identification with scores on mental health assessment tools. PCPs recognized MHPs in 26–60% of the children with elevated scores on assessment tools (for purposes of simplification further indicated as ‘correct’ identification) [21–23,32,36,38,42,44–46,48–50]. Seven studies investigated factors associated with ‘correct’ identification, though most studies only investigated one factor. PCPs more often identified children with an increased risk of MHPs when children were older, were boys, well-known to their clinician, were visiting for a psychosocial problem, when PCPs used an assessment questionnaire such as the Child Behavior Checklist (CBCL) or when PCPs were trained in MHP recognition [34,38,46,48]. Practice type, ethnicity, family composition, PCP work experience and parent-reported concerns showed no consistent association with ‘correct’ identification [32,34,38,45,46,48]. One study found that physicians experiencing a higher MHP burden identified fewer children with problems as evaluated by mental health assessment tools, but identified more children in whom assessment tools did not indicate MHPs [35].

## Discussion

### Main findings

This study presents the results of a systematic review of literature regarding factors associated with the identification of child MHPs by PCPs. Most of the included studies were performed in the US and the Netherlands. Prevalence rates of identified MHPs varied between studies and PCPs recognized 26–60% of children with an elevated score on MHP screening tools. Overall, we found that MHPs were more often identified among children with mental health symptoms, with a family composition other than married parents and with a history of MHPs. Boys in junior school and children who visited a PCP regarding psychosocial concerns or a well-child visit were also more often identified with a MHP. PCPs who felt less burdened treating MHPs and PCPs recently trained in child MHPs were more likely to identify MHPs and also more likely to recognize MHPs in children showing an increased score on MHP assessment tools. Interestingly, discussion of MHPs during a consultation only resulted in more PCP-identified MHPs when the exploration was reported by PCPs, but not when parents reported the exploration. No clear association was found between other background characteristics of child, family, and professionals and PCP identification of child MHPs.

### Comparison with previous reviews

In line with reviews by Zwaanswijk et al. [26] and Sayal et al. [25], published over a decade ago and based on fewer studies, our study confirms the association of the factors family composition, past treatment for MHPs, severity of child psychopathology, mental health symptoms, type of visit, professional acquaintance with the child, professional training, parental expression of concerns with the identification of child MHPs by PCPs. In addition, we found that prior life events led to more MHPs identified only during school age [19,23,31–38,42,44,47,49–51]. Zwaanswijk et al. and Sayal et al. [25,26] included fewer studies reporting on this association and did not mention a difference in the association between prior life events and MHP identification across ages.

Sayal et al. [25] also reported that other factors preventing GPs from recognizing or dealing with mental health issues are likely to reflect lack of confidence, skills, or knowledge. This is in line with our findings that PCP identification was influenced by the PCP’s psychosocial orientation and the PCP’s experienced burden treating MHP.

In contrast to Zwaanswijk et al. and Sayal et al. [25,26], our study did not confirm the association between male gender and increased MHP identification across all ages. Our study showed that male gender was only associated with increased identification at junior school age, a finding that may be related to the fact that boys have higher rates of problems and that MHPs become more apparent at the age when a child enters the school setting [3,49]. In addition, we did not find a clear association between a child’s age and MHP identification. Zwaanswijk et al. [26] reported a clear association between older age and MHP identification, while Sayal et al. [25] only reported a similar result in studies performed in both preventive and curative care or in curative care only. However, Sayal et al. [25] found that a younger age was associated with MHP identification in one study performed in preventive care only [25]. In our study, the study setting did not impact the association between age and MHP identification. Also, we did not find an association between limited service availability to refer patients to and a decreased MHP identification.

The number of MHPs identified by PCPs varied between studies, with lower rates found in studies involving younger children. More importantly, however, we found that identification rates varied between similar professionals within studies [37,46,47]. This variance could not be explained by child characteristics [37,46] and could only be partly explained by the included PCP or practice characteristics [37,46,47]. Nevertheless, a large part of the variation in identification rates remained unexplained, suggesting that other factors in the recognition process play a role. To improve the identification of child MHPs, and decrease the inter-professional variation in identification, we suggest that the knowledge gap explaining the inter-professional variation should be targeted in future studies. For instance, good professional training and the use of protocols have shown to reduce inter-professional variation and improve the identification of problems in children showing elevated scores on MHPs assessment tools [20,48]. Proper professional training is also likely to influence positively the PCP’s focus on psychosocial well-being and PCP experienced burden treating MHPs, factors that were reported to impact PCP identification of child MHPs in our study. The importance of training and skills was also confirmed by PCP-reported barriers to the identification of MHPs [14,52–55]. However, it should be taken into account that training activities may be time-consuming and that training activities may only improve MHP identification in the short-term [20,48].

The identification of MHPs was related to the number of mental health symptoms and a history of problems, both signifying more severe problems [19,34,35,37,42,44,46,47,49,50]. Parental disclosure of mental health concerns only resulted in higher identification rates when professionals recognized that parents had raised concerns [21,22,50]. Parents might fail to disclose their concerns effectively [39], and professionals often do not agree with parent-reported concerns or that psychosocial information was discussed during consultation [22]. Other explanations might relate to professionals not adequately responding to parental disclosure or to other as yet unknown factors in the recognition process.

### Strengths and limitations

We used a wide-ranging search strategy in leading medical and psychological databases and in the grey literature to avoid overlooking relevant articles. This approach expands on two prior reviews which used relatively short search strategies limited to either two or three databases [25,26].

An important feature of this review was the inclusion of studies performed in both preventive care and curative care. Although healthcare systems worldwide vary considerably, a preventive healthcare programme for children can be found in most countries, and primary care attendance rates are consistent among different healthcare systems [10,56,57]. The inclusion of studies from both settings also provided broader information on factors associated with the identification of child MHPs by professionals in primary care. While not all factors were investigated in studies of both preventive and curative care, factors that were investigated in studies that included both settings generally showed similar associations when compared to studies performed in only one setting.

Unfortunately, most studies did not include an independent assessment of the child’s mental health, e.g. by a questionnaire such as the CBCL. PCP recognition differed between professionals, so some PCPs appear more inclined to identify MHPs than others. It is also possible that some PCPs were more focussed on reporting MHPs in specific children, e.g., in children with divorced parents. Therefore, the associations found in our study do not necessarily predict actual MHPs. Future studies should compare factors associated with PCP-identified MHPs and factors associated with objectively proven MHPs.

In addition, most studies did not define the term child MHPs. This may have impeded the comparison of study results and might (partly) explain the wide variation in identification rates. The included studies, however, reflect the identification process as found in daily practice and most studies measured identification by asking the professional whether they thought a MHP was present, indicating the investigation of a broad concept of MHPs, which corresponded with the aim of our study [20–23,31–35,37–40,42–50].

Additionally, in this review, we only presented results after adjustment for several background variables. As the included studies adjusted for different sets of background variables, this probably hampered comparability of the studies. In studies that also reported univariable analyses, the univariable results did not alter conclusions based on multivariable results.

### Implications

Some characteristics were investigated in only one study, while the identification of MHPs indicated by mental health assessment tools was investigated in relatively few studies. An increased risk flagged by MHP assessment tools only indicates that a child might experience problems and that further attention is warranted, it does not imply a MHP diagnosis. To obtain more robust evidence regarding factors associated with PCP-identified MHPs, and especially the identification of children with an increased risk of MHPs, we recommend better exploration of factors determining identification of child MHPs by PCPs.

In addition, further insight into the factors explaining variations in MHP identification is needed. This could be facilitated by a study design in which the actual identification process is monitored. The next challenge is to decrease variation in identification and to ensure that the right children are identified. Training and screening tools might increase the sensitivity of professionals (and decrease variation) but might also lead to an increase in the number of children identified and thus to more ‘false positives’ needing additional assessment [58]. An understanding of the factors associated with missed MHP identification in children flagged by independent mental health assessment is important to the framing of strategies and policies to improve identification. In this review, we identified relatively few studies investigating this problem. As mentioned above, we recommend that this issue should be targeted in future studies. Combining data from different sources, including data from routine healthcare, might have great potential for improving MHP recognition [59]. For example, in the Netherlands each child participates in regular preventive health assessments performed in community paediatric centres, thus providing a long-term overview of the child’s health status. Additionally, a general practitioner is usually consulted when children or parents have health problems and can, therefore, monitor family developments and possible effects on a child’s health [19,56]. Combining complementary information from different sources might aid better problem identification.

## Conclusion

MHPs were more often identified in children with more mental health symptoms, with prior MHPs, among boys in junior school or as a result of visits to PCPs related to psychosocial concerns or well-child visits. In addition, PCPs who felt less burdened treating MHPs and PCPs who were recently trained in child MHPs were more likely to identify MHPs, and more likely to recognize MHPs in children with an increased score on MHP assessment tools. Factors associated with PCP-identification of children with an increased risk of MHPs were largely comparable with factors associated with MHP identification in general, but were investigated in relatively few studies.

## Data Availability

All data generated or analysed during this study are included in this article or in supplementary information files.
